# Bone Type Selection for Human Molecular Genetic Identification of Skeletal Remains

**DOI:** 10.3390/genes16080872

**Published:** 2025-07-24

**Authors:** Jezerka Inkret, Irena Zupanič Pajnič

**Affiliations:** Institute of Forensic Medicine, Faculty of Medicine, University of Ljubljana, Korytkova 2, 1000 Ljubljana, Slovenia; jezerka.inkret@mf.uni-lj.si

**Keywords:** skeletal elements sampling strategy, DNA content in bones, DNA quality, DNA quantity, molecular genetic identification

## Abstract

This review paper presents a comprehensive overview of DNA preservation in hard tissues (bones and teeth) for applications in forensic and archaeogenetic analyses. It presents bone structure, DNA location in bones and teeth, and extensive information about postmortem DNA location and preservation. Aged bones are a challenging biological material for DNA isolation due to their low DNA content, degraded DNA, and the potential presence of PCR inhibitors. In addition, the binding of DNA to the mineral matrix necessitates the inclusion of a demineralization process in extraction, and its contribution to the resulting increase in both DNA quality and quantity is explained. Guidelines and recommendations on bone sample selection to obtain higher DNA yields are discussed in terms of past, recent, and possible future recommendations. Interskeletal and intraskeletal differences in DNA yield are also explained. Recent studies have shown that current recommendations for the genetic identification of skeletal remains, including femurs, tibias, and teeth, may not be the most effective sampling approach. Moreover, when mass disasters and mass graves with commingled skeletal remains are considered, there is a greater possibility that the recommended set of skeletal elements will not be available for sampling and subsequent genetic testing. This review highlights interskeletal and intraskeletal variability in DNA yield, with a focus on studies conducted on poorly preserved skeletal remains, including both postwar (1945) victims from Slovenia and ancient human skeletons. Special emphasis is placed on anatomical differences and potential mechanisms influencing DNA preservation, as demonstrated in research on both modern and historical skeletons. Finally, the petrous part of the temporal bone and tooth cementum were reviewed in greater detail because they have been recognized as an optimal sampling type in both ancient DNA studies and routine forensic case analyses. Our experiences with the Second World War and archaeological petrous bones are discussed and compared to those of other bone types.

## 1. Introduction

Skeletal remains, particularly bones and teeth, represent the most durable biological evidence in postmortem interval and retain genetic material suitable for individual identification. DNA persistence in bone is attributed to its adsorption to hydroxyapatite and collagen within the mineralized matrix [1,2,3]. The matrix comprises inorganic hydroxyapatite and organic components, primarily type I collagen and various non-collagenous proteins [4], both of which contribute to DNA stability and influence extraction efficiency. These interactions are critical for optimizing demineralization-based recovery protocols [5].

Lindahl and Okazaki were among the first to propose that DNA preservation in bone is primarily associated with its inorganic component, specifically hydroxyapatite [2,6]. DNA stability within bioapatite crystals is attributed to electrostatic interactions between calcium ions and the phosphate backbone, as well as binding to hydroxyl sites on hydroxyapatite [2,7,8]. This mineral association reduces DNA depurination compared to unbound DNA [2]. Two mechanisms have been proposed for this binding: (1) in vivo adsorption and encapsulation during bone growth and remodeling, and (2) postmortem binding of DNA fragments to hydroxyapatite within bone micropores during mineral re-precipitation following cellular degradation [5].

Given this article’s focus on postmortem DNA preservation in bone, it is essential to consider the four primary pathways proposed by Campos et al. [5]. Two mechanisms occur during life: (1) DNA binds to collagen fibrils and is subsequently encapsulated during hydroxyapatite mineralization, and (2) DNA associates with or becomes entrapped by hydroxyapatite crystallites forming within interfibrillar spaces during osteoid mineralization. Postmortem, two additional diagenetic pathways are proposed: (1) DNA adsorbs onto exposed collagen fibrils within bone macroporosity following chemical or microbial degradation, and (2) DNA binds to or becomes encapsulated by re-precipitating hydroxyapatite during mineral recrystallization processes.

Alternatively, DNA may be preserved within the organic matrix of bone through interactions with collagen, either by forming protein-DNA complexes, becoming entombed within the matrix, or via covalent cross-linking to proteins [9,10]. Collagen is thought to stabilize the DNA double helix and its hydration shell via hydrogen bonding, thereby enhancing molecular stability and prolonging postmortem DNA survival [10,11,12].

Subsequent work demonstrated that mineral binding plays a more critical role. Kleter et al. and Putnis and Putnis [13,14] showed that hydroxyapatite mineral degradation progresses gradually in the presence of organics, but once the mineral matrix deteriorates, collagen becomes exposed to enzymatic breakdown, accelerating tissue decay. Götherström et al. [15] further established a strong correlation between DNA preservation and hydroxyapatite crystallinity in both experimentally degraded modern and archaeological bones, highlighting the mineral phase as the key determinant. More recent work by Campos et al. [5] supports this view, emphasizing the role of the mineral-organic matrix, where collagen contributes structurally but hydroxyapatite is primarily responsible for long-term DNA stability.

## 2. DNA Recovery from Hard Mineralized Tissues

Advancements in understanding DNA localization in postmortem hard tissues paralleled the emergence of ancient DNA (aDNA) studies. Initial aDNA recoveries were from archaeological and museum-preserved animal soft tissues rather than bone [16,17,18,19]. The first aDNA sequence was obtained from a 140-year-old skin sample of an extinct quagga, demonstrating DNA survival in soft tissue under favorable conditions [16]. Bone-derived aDNA was first recovered by Hagelberg in 1989 [1], who later extracted DNA from 16th-century porcine bone [20] and modern human bone in a forensic case [21].

Hochmeister et al. [22] were the first to extract nuclear DNA from a human femur with an 18-month PMI. They later used commercial multiplex PCR kits to identify skeletal remains recovered after one year [23]. Femurs played a crucial role in identifying the Romanov family, exhumed after 75 years [24]. DNA was also successfully extracted from vertebrae [25,26]. Subsequent studies reported variable success in DNA recovery from bones with differing PMIs [27,28,29].

DNA recovery from bones and teeth is challenging due to the hard mineralized matrix, requiring specialized equipment and contamination-controlled environments [30]. Bone and tooth extracts contain PCR inhibitors such as calcium and collagen, necessitating their removal to optimize amplification [31]. DNA adsorbs strongly to hydroxyapatite and collagen, making demineralization essential for maximal recovery [2,8,32]. Early protocols using EDTA-based demineralization increased DNA yield [20], with subsequent modifications enhancing STR profile quality from bones aged 5–100 years [32]. Comparative analyses of DNA extraction methods have demonstrated that the use of fine bone powder, combined with EDTA-based decalcification and proteinase K digestion, significantly enhances both DNA yield and quality, even from minimal input material [33,34]. These results have been consistently corroborated by subsequent studies [35,36,37]. Among more recent advances, the Dabney extraction protocol—originally developed for aDNA applications—has proven highly effective in forensic settings as well, enabling the recovery of ultrashort DNA fragments (≥35 bp) from highly degraded skeletal material and thus maximizing the recovery of endogenous DNA [38]. A recent study even showed that a protocol with incomplete decalcification, described by Dabney [38], is more effective in recovery of shorter DNA molecules [39].

Building on these advancements, our research group has adopted a complete demineralization protocol based on Zupanič Pajnič [40], which has shown robust performance across a wide range of sample types, including Second World War mass grave victims, modern forensic cases, and archaeological remains from Slovenia [41,42,43].

## 3. Recommendations for Bone Sampling Strategies

Bone sampling recommendations for disaster victim identification (DVI) have evolved based on empirical findings and institutional guidelines. The earliest structured recommendations were issued by the International Society for Forensic Genetics (ISFG) in 2007, emphasizing the collection of long, dense, weight-bearing bones—particularly femora—and healthy, unrestored teeth as primary targets for DNA analysis [44].

Subsequently, additional general guidance was provided by Interpol and the U.S. National Institute of Justice, which outlined procedures for bone and tooth sampling but did not specify a hierarchy of skeletal elements [45,46]. A more refined approach was introduced by the International Commission on Missing Persons (ICMP) in 2015, which offered explicit prioritization of skeletal sampling sites: healthy teeth first, followed by femur, tibia, and pelvis [47]. These guidelines, based on Hines et al.’s study of remains with 18–21-year postmortem intervals (PMIs) from the Western Balkans, rank skeletal samples as teeth, tali, other tarsals, petrous bone, femurs, vertebrae, tibias, and metatarsals. Despite high DNA yields from metatarsals, femurs remain preferable for commingled remains due to easier reassociation of larger bones [48].

Earlier empirical evidence supporting these recommendations was provided by Edson et al. [49], who reviewed DNA analysis results from 1021 skeletal remains processed by the Armed Forces DNA Identification Laboratory (AFDIL) under diverse environmental conditions. Their findings confirmed that dense, weight-bearing long bones—particularly femora and tibiae—consistently yielded the highest DNA quantities. Metatarsals and ribs also performed relatively well, while cranial bones showed the poorest outcomes, confirming that porous bones are less suitable for DNA extraction compared to dense cortical bones.

Building on established guidelines prioritizing weight-bearing bones for DNA sampling, several large-scale studies have further validated these recommendations. Miloš et al. [50] analyzed 25,361 skeletal elements, revealing significant interskeletal variation in DNA yield, with femurs and teeth providing the highest DNA quantities, while clavicles, ulnae, and radii yielded the least. Similarly, post-9/11 (World Trade Center disaster in 2001) identification efforts involving 2749 victims demonstrated that lower limb bones—excluding the fibula—yielded more DNA than upper limb or axial skeleton elements. Interestingly, the patella, metatarsals, and foot phalanges surpassed the femur in DNA yield, highlighting their value as alternative sampling sites [51].

Although small cancellous bones are good DNA sources, long, dense, weight-bearing bones—especially the femur—remain the preferred samples [44]. This is due to reduced DNA degradation in dense bone [52,53] and the ease of reassociating larger skeletal elements in commingled remains.

The effectiveness of femora and teeth in DNA-based identification was demonstrated in several Second World War case studies from Slovenia. High identification rates were achieved even from fragmented femurs and tibias, confirming their value in degraded remains and supporting their prioritization in forensic sampling strategies [41,42,43].

Alongside long bones, teeth have proven to be reliable sources for DNA typing, especially in Second World War victims. Teeth are recommended as primary sampling material in DVI guidelines and are often favored or considered comparable to the petrous bone [41,54]. Studies show high DNA yields from teeth in both forensic and ancient samples [55,56,57].

Teeth’s unique structure and position in the jaw protect DNA from microbial and environmental degradation [49,58]. Different tooth tissues—pulp, dentin, and cementum—vary in DNA content both between and within individuals [59,60]. Pulp is a good DNA source in healthy, fresh teeth but degrades quickly with disease, age, and postmortem decay [61,62]. Environmental factors influence pulp preservation; mummification aids its survival, while putrefaction destroys it rapidly [63,64].

Besides pulp, dentin and cementum also contain DNA. Dentin yields nuclear and mitochondrial DNA, but amounts decrease with age and disease [60]. Cementum is increasingly recognized as a reliable DNA source, especially when pulp is absent [56,62]. Studies show cementum contains nucleated cells and yields successful STR profiles [57]. Unlike dentin, cementum resembles bone but differs by being avascular, non-remodeling, and thickening over time [65,66].

Sampling cementum offers several advantages: minimal contamination risk, simplified extraction, small sample size needed (15–50 mg), and no need for full demineralization. Cementum has been shown to preserve DNA longer than other dental tissues [67].

Recent advances in DNA extraction from teeth have introduced less invasive alternatives to traditional whole-tooth grinding and decalcification methods. New protocols include direct access to the pulp cavity via tooth opening [68] and targeted sampling of the root cementum, which has been shown to contain higher concentrations of endogenous DNA compared to dentin [69,70,71]. To further enhance DNA yield, some studies have proposed the combined sampling of dentin and cementum [72].

Recent studies challenge the traditional recommendation of weight-bearing compact long bones as the best DNA source, highlighting small cancellous bones as superior samples. Notably, Mundorff’s large study of 3631 skeletal samples from the World Trade Center disaster found the highest DNA yields in foot phalanges and patellae, followed by metatarsals, femurs, and tibias [51]. However, earlier studies were retrospective, lacked control for environmental factors and PMIs, and did not sample all representative skeletal elements. Mundorff’s 2014 study was the first to analyze all major skeletal elements under uniform PMI and environmental conditions [73]. This was followed by two additional studies with similar design, also assessing comprehensive skeletal sampling under controlled conditions [74,75].

Mundorff’s 2014 study analyzed 55 skeletal elements from 3 recently skeletonized individuals and 120 bones from 12 skeletons with varying PMIs, all exposed to the same environmental conditions and geographic location [73]. Results showed that small bones of the hands and feet (metacarpals, metatarsals, and phalanges) yielded more DNA, while femurs and tibias ranked among the ten lowest-yielding elements [73].

Emmons et al. [74] analyzed 49 skeletal elements per individual from 3 recently buried skeletons to assess DNA yield across all bone types. Foot bones consistently yielded the highest DNA quantities, with hand bones ranking second in two cases. Notably, no long bones were included in the top ten.

A similar study by Zupanc et al. [75] examined 144 bones from 3 Second World War skeletons exhumed from the same mass grave, ensuring uniform PMI and environmental exposure. The third metacarpal consistently produced the highest DNA yield. Based on DNA quantity and STR typing success, the top-ranking elements were metacarpals, metatarsals, and the petrous portion of the temporal bone. Elements with consistently poor results included the ulna, clavicle, radius, scapula, humerus, and various cranial and pelvic bones. These findings highlight the utility of small cancellous bones and the petrous bone as superior sampling sites for DNA analysis.

The recently performed studies described above came to the same conclusions, showing that small skeletal elements outperform the currently recommended weight-bearing long bones. It is important to note that the studies, including many anatomically different skeletal elements that represent all representative bone types in the human body, have always shown the usefulness of small skeletal elements. Different ranking orders of the most appropriate skeletal elements vary from study to study, but they all show the usefulness of the small skeletal elements of the hands and feet. Here it must be pointed out that older studies were all retrospective [49,50,51,76] and did not include all anatomically representative skeletal elements, the same PMI, and similar environmental conditions. In older studies, the femur is often ranked first or among the highest-ranking skeletal elements, but even older studies showed the potential of small cancellous skeletal elements. Metatarsals were fifth among the highest-ranking elements in a study by Edson [49] and third in a study by Leney [76], and foot phalanges, patellae, and metatarsals outperformed femurs and tibias in the World Trade Center DVI study [51]. An overview of key studies on skeletal element selection for DNA analysis, outlining the number of elements tested, PMIs, and the highest-yielding bones, and contrasts traditional guidelines with recent findings that propose alternative sampling strategies, is presented in Table 1.

To refine sampling strategies and formulate bone selection guidelines for the genetic identification of postwar victims in Slovenia, our research group used the Konfin Shaft II mass grave as a model of poorly preserved remains [78]. This approach ensured that all bones had been subjected to the same PMI and environmental conditions. Up to 43 skeletons were analyzed to evaluate inter-bone variability in DNA preservation. A total of 566 skeletal elements were categorized into 7 anatomically and morphologically defined groups: the temporal bone group; long bones (femur and tibia); torso bones (first ribs and 12th thoracic vertebrae); metacarpals; metatarsals; short and sesamoid bones (including the talus, navicular, medial cuneiform, cuboid, calcaneus, and patella); and teeth [78]. DNA yield and quality varied significantly across these groups. The highest DNA concentrations were found in the petrous part of the temporal bone, metacarpals, torso bones, and short/sesamoid bones, with no statistically significant differences in DNA quality among them, despite their structural differences. In contrast, long bones, metatarsals, and teeth yielded lower DNA amounts, with metatarsals outperforming long bones, and teeth exhibiting the lowest overall DNA content [78].

Based on these findings, our research group developed the Slovenian guidelines for sampling skeletal elements in Second World War mass grave investigations, aligned with the third ISFG recommendation [44] and emphasizing a multi-sample strategy. In addition to femurs, tibias, and teeth—recognized by ISFG and ICMP [44,47] as primary targets—bones from the metacarpal, short and sesamoid, and torso groups are also recommended when complete skeletons are available. These elements not only provide high DNA yields but are also quick and easy to sample, even with a scalpel. In cases of commingled remains, anatomically distinctive and easily recognizable bones—such as the petrous part of the temporal bone, femurs, tibias, first ribs, 12th thoracic vertebrae, and large tarsals like the talus and calcaneus—should be prioritized. The petrous bone is especially important when only cranial remains are present [78].

When evaluating skeletal elements in routine forensic casework involving short PMIs—ranging from approximately 1 month to 21 years—under varying environmental and physical conditions, the small cancellous bones of the hands and feet demonstrate consistently higher DNA yields compared to cortical bones [77,79]. Notably, this trend persisted regardless of PMI duration, and even in cases with short PMIs (≤1 year), cancellous bones preserved significantly greater quantities of DNA. These findings highlight the value of small cancellous bones as viable alternatives to traditionally preferred elements such as long bones and teeth. Given their high DNA recovery potential, ease of processing, and suitability for degraded remains, these bones should be actively considered for efficient and reliable human identification in forensic genetics [77,79].

However, small skeletal elements are more prone to degradation with increasing PMI, and they are more easily lost or missed during excavations [44]. The same is true for burned remains, where distal and low-density bones—such as phalanges, metacarpals, and tarsals—are frequently destroyed or rendered unsuitable for DNA sampling due to their small size, low density, limited soft tissue protection, and high susceptibility to thermal damage and mechanical fracturing [80]. In such cases, the petrous portion of the temporal bone represents the most promising alternative. Owing to its high density and protected location within the skull, it has shown superior resistance to thermal damage and consistently yields significantly higher quantities of endogenous DNA—up to 4 to 16 times more than teeth—even after severe burning [54,69,80,81]. Misner et al. [82] examined whether the degree of visible weathering in bone correlates with mitochondrial DNA yield and quality. Their results indicated that, despite weathering potentially reducing the amount of viable sampling surface, the macroscopic appearance of thermally altered bone does not reliably reflect the extent of DNA preservation.

Nevertheless, we recommend sampling small skeletal elements whenever possible, together with traditionally recommended long weight-bearing bones, which tend to remain present even with a longer PMI. Small skeletal samples have many advantages in addition to producing a higher DNA yield. Their collection is easier, quicker, and safer, and no additional equipment is needed. The sampling procedure is thus shortened and less prone to possible contamination. In mass fatality events, collecting and properly storing multiple skeletal element types from the beginning of sampling makes it possible to avoid laborious re-sampling and re-labeling efforts, and sampling of multiple skeletal element types also follows the third ISFG recommendation [44]. Although developed within a forensic framework, these sampling recommendations are also applicable in archaeogenetic contexts, where recent studies have shown that elements such as the calcaneus and talus, like the petrous bone, can yield well-preserved DNA despite extended PMIs and varied burial conditions [83].

## 4. Intrabone Variability in DNA Content

Intra-element variability in DNA yield has been investigated in several studies, some of which focused on non-human remains [84,85,86], and others on human teeth and bones [67,74]. All studies reported notable differences in DNA yield between anatomical regions within the same skeletal element.

Barta et al. [84], analyzing ~3000-year-old fur seal ribs, also found substantial intra- and intersample mtDNA variation, with no clear correlation between bone density and DNA preservation. Antinick et al. [86] conducted 3200 assays on fresh and environmentally exposed bovine and porcine femurs, calcanei, and tali. In both conditions, the femoral epiphyses yielded more DNA than mid-diaphyseal regions, while tarsals produced intermediate yields.

In contrast, other studies found denser bone regions to yield more DNA. Alberti et al. [85] observed significantly higher endogenous DNA recovery from dense cortical layers in ancient cave bear and leopard long bones and petrous bones—up to 29-fold more than trabecular regions. However, the outer bone layers are often removed during sampling to reduce contamination.

Emmons et al. [74] assessed intrabone variability in 49 bones from 3 modern human skeletons, sampling multiple sites within selected bones. The femur, humerus, and tibia showed the greatest differences, with higher DNA yields consistently in the proximal ends (e.g., femoral head) compared to midshaft or distal regions.

A study by Klavens et al. [87] compared five regions in fresh human femurs and tibias, reporting the highest DNA yields and STR typing success in the mid-diaphysis, and lowest yields in the proximal diaphysis. Unlike previous studies, this work found central diaphyseal regions to be most suitable, though it excluded epiphyseal sampling and was limited to fresh remains (PMI: hours), making direct comparison with older remains difficult. In contrast, our study on World War II femora showed that the femoral head, rich in trabecular bone, yielded higher DNA quantities and is thus a more suitable sampling site for degraded remains [88].

To investigate intraskeletal DNA yield variability in World War II remains, our group analyzed 193 metatarsals and metacarpals from a single mass grave, ensuring consistent PMI and environmental conditions [89]. Previous work by our team identified metacarpals and metatarsals as among the most DNA-rich skeletal elements [75]. Structurally, these bones (metatarsal and metacarpals) consist of a compact diaphysis and cancellous epiphyses [90]. For each bone, diaphyseal and epiphyseal regions were sampled separately. DNA yields were significantly higher in the epiphyses compared to the diaphyses in all bones tested, with no difference in DNA quality [89]. Thus, cancellous bone in the epiphyses is the preferred sampling site. Among all samples, the epiphysis of the third metacarpal yielded the highest DNA concentration, making it the most suitable sampling target. Metacarpals should be prioritized over metatarsals when both are available. This contrasts with Emmons et al. [74], who found no significant intraskeletal differences in fresh remains. Our findings emphasize the practical relevance of sampling epiphyses over diaphyses in degraded historical remains.

Intraskeletal variation in DNA yield has also been demonstrated in elements such as the first rib and thoracic vertebrae. Sampling of different regions of the first rib revealed significantly higher DNA quantities in the proximal segment compared to the middle and distal parts, likely due to greater bone remodeling and mechanical loading [91]. Similarly, analysis of the 12th thoracic vertebra showed the highest DNA yield in the laminae and spinous process, followed by the pedicles, with the vertebral body yielding the least DNA [92]. These findings emphasize the importance of selecting anatomically favorable regions within bones to optimize DNA recovery.

## 5. Differences in DNA Preservation in Compact and Trabecular Bone

Recent studies have consistently shown that trabecular (cancellous) bone preserves significantly more DNA than compact (cortical) bone, highlighting critical differences in DNA preservation across bone microstructures [73,74,75,89]. One of the main explanations lies in the porous architecture of trabecular bone, which protects residual soft tissues from rapid diagenetic degradation. Andronowski et al. [93,94], using synchrotron radiation micro-CT, demonstrated that cancellous bone—especially in epiphyseal regions—often retains soft-tissue remnants such as marrow, periosteum, and bone-lining cells. These tissues likely contribute to higher DNA yields. Additional support for this hypothesis comes from X-ray photoelectron spectroscopy analyses showing higher carbon content and lower mineralization in trabecular bone compared to compact cortical regions (Andronowski et al. 2019) [95].

Molecular differences between trabecular and cortical bone were studied using spectroscopic techniques [96,97]. ATR-FTIR spectroscopy, widely used in DNA preservation research [98,99,100,101,102,103], showed higher organic content in epiphyses and more carbonates and phosphates in diaphyses of hand and foot bones [104], supporting soft tissue’s role in more DNA preservation in cancellous and epiphyseal parts of bones.

Earlier work by Schweitzer et al. [105,106,107] similarly reported the presence of soft-tissue structures in trabecular regions of archaeological animal bones, observed via scanning and transmission electron microscopy. These structures included preserved blood vessels, fibrous matrix, and osteocytes, although alternative explanations—such as microbial biofilms or kerogenized residues—were also proposed. While some researchers [108] have questioned whether these structures represent authentic soft tissues or diagenetic products, the consistent detection of organic material within trabecular compartments supports their relevance in explaining enhanced DNA preservation.

A second hypothesis for higher DNA yields in cancellous bone is bacterial infestation. Kaye et al. [109] identified bacterial biofilms in cancellous bone via electron microscopy. These biofilms persist long-term due to mineralization protection. Porous bones with trabecular spaces show increased bacterial presence [15]. Some bacteria degrade organic bone but spare DNA [110]. Emmons et al. [111] found higher bacterial loads in cancellous bone from decomposed human remains, linked to soft tissue remnants [93]. They concluded that microbial presence correlates positively with DNA yield and may aid DNA preservation [111]. However, bacterial colonization also raises the potential for microbial DNA contamination, complicating the accurate detection and quantification of authentic human DNA. Thus, the role of bacterial presence in DNA preservation and its possible interference with forensic DNA analysis warrants careful consideration and further investigation. However, increased microbial presence also raises concerns about potential contamination with non-human DNA, which can interfere with the accurate recovery and interpretation of endogenous human genetic material. To address this, various pretreatment protocols have been developed to reduce exogenous microbial DNA before sequencing. For example, phosphate buffer washes and sodium hypochlorite treatments have been shown to significantly decrease microbial DNA content while variably preserving endogenous DNA, thereby improving the proportion of informative sequences [112,113]. Nonetheless, the complete removal of contaminating DNA remains a persistent challenge, especially in highly degraded or microbially colonized skeletal material.

Intraskeletal differences in DNA yield may reflect variability present during an individual’s lifetime, though this cannot be verified due to the impossibility of bone sampling in living persons. Such DNA content variation across bones may be static or fluctuate throughout life [86]. Bone develops by replacing collagenous mesenchymal tissue with primitive bone, which remodels into mature bone that grows over time. Epiphyseal plate closure marks the end of bone growth and correlates with biological age [114,115,116]. This could explain higher DNA yields in distal bone parts of younger individuals, though this hypothesis remains untested as most archaeological and forensic studies lack detailed age analyses.

Differences in DNA content may also result from bone remodeling, explaining intrabone heterogeneity and higher DNA yields at articulation or muscle attachment sites, as well as in bones with higher remodeling rates [86]. Local mechanical stress stimulates bone cells to adjust structure for mechanical loading [117], with about 30% of remodeling targeting specific sites [118]. Epiphyses, which distribute body weight (e.g., femoral head) and aid locomotion (small cancellous bones), are likely such sites [119]. Muscle attachment sites may also undergo remodeling, contributing to increased DNA yields observed in various studies [73,74,75,86]. Leney emphasized remodeling’s role, recommending femurs and tibias for DNA sampling due to their high mechanical stress from body weight and movement [76].

Differences in DNA preservation between compact and trabecular bone were systematically investigated in the study comparing full dissolution (FD) and partial dissolution (PD) methods for DNA extraction from compact and trabecular bone, revealing that FD was more effective only in compact bone, while PD yielded comparable results in trabecular bone. Notably, trabecular bone produced DNA of equal or even better quality than compact bone, likely due to residual soft tissues and its higher metabolic activity during life. These findings suggest that different extraction protocols should be tailored to bone type, with FD preferred for compact bone and PD sufficient for trabecular bone, thereby improving efficiency and reducing contamination risk. However, due to its greater vulnerability to environmental degradation, trabecular bone may be less reliable than compact bone for aDNA recovery in cases of longer PMI [88].

## 6. The Petrous Bone and Tooth Cementum

Studies on postcranial skeletal elements and teeth have identified the petrous bone (pars petrosa of the temporal bone) as the most promising source for DNA extraction [120]. Edson [121] reported a 90% success rate for recovering mitochondrial DNA from the temporal bone, with later studies confirming the petrous portion as the optimal sampling site due to its reduced exposure to weathering [82]. The petrous bone is the hardest mammalian bone and develops differently from other skeletal bones [122]. Its vestibulo-cochlear organ is encased by the otic capsule, formed by endochondral ossification starting in utero around the 16th gestational week and completed by birth [123,124]. This primary fetal structure remains unchanged throughout life, with no remodeling in the inner otic capsule layers after two years of age [122,125,126]. The petrous bone’s dense structure and limited vascularization likely reduce microbial infiltration and biochemical degradation that harm DNA preservation [54]. Additionally, it has a relatively high density of osteocytic lacunae and osteocytes compared to bones like the femur [127], providing a reservoir for nuclear material and contributing to high DNA yields. Although DNA from the petrous bone often shows degradation and lower molecular integrity, its total DNA yield remains consistently high, indicating unique histological and microstructural features that support DNA preservation even when quality is compromised in other bones.

The petrous portion of the temporal bone is widely recognized as the most reliable skeletal element for aDNA analysis, consistently yielding significantly higher amounts of endogenous DNA compared to other bones. It can contain up to 16 times more DNA than teeth and up to 183 times more than bones such as ribs, metacarpals, or metatarsals [81]. Studies have shown that even when degraded, DNA from the petrous bone produces superior STR profiles than that from femurs or teeth [128,129]. This exceptional preservation is largely attributed to the otic capsule—the densest part of the human skeleton—which undergoes minimal postnatal remodeling and provides a stable microenvironment for DNA [125,130]. Furthermore, Hansen et al. [69] demonstrated that the petrous bone outperforms even tooth cementum in retaining endogenous DNA under adverse preservation conditions.

A series of aDNA studies conducted by our research group—predominantly utilizing well-preserved petrous bones—has demonstrated their exceptional capacity for DNA preservation, even in highly degraded or juvenile skeletal remains [131]. The genetic analysis of skeletons from the Bled–Pristava grave site further confirmed that petrous bones yield superior STR typing results compared to teeth, enabling reliable molecular sex identification, including subadult individuals [131]. Our research group established a comparative baseline for evaluating how environmental variables such as temperature, humidity, and soil chemistry impact DNA integrity across archaeological sites, revealing site-specific differences in DNA yield and degradation [132]. Furthermore, our findings show that long-term storage under uncontrolled conditions—especially fluctuating temperature and humidity—significantly compromises DNA quality, emphasizing the need for regulated environmental storage to preserve genetic material [133].

Beyond archaeology, the petrous bone is valuable in forensic identification, as shown by Kulstein [134] and in older forensic cases from the Second World War [135].

Gonzales et al. [54] comprehensively demonstrated the advantage of petrous bone sampling for forensic STR typing through systematic analysis of 65 petrous bones and 50 teeth. Their study also examined the petrous bone’s histology, confirming the presence of glycoproteins in the inner ear’s border region of highly degraded archaeological samples. The persistence of these glycoproteins (cartilaginous matrix) indicates the otic capsule’s isolation from other skeletal elements and distinct biochemical processes, as glycoproteins degrade rapidly elsewhere [54]. Histological analysis revealed chondrocyte remnants—markers of immature bone—in this region, consistent with sparse vascularization and absent remodeling reported by Pinhasi et al. [130]. Thus, the otic capsule is largely excluded from vascularization and potential microbial contamination [54].

Our research compared DNA yield and STR typing success between 26 petrous bones and 30 metacarpals III from a Second World War mass grave [136]. Metacarpals III were selected based on previous findings because they yield the highest DNA among skeletal elements [75,89]. We found no statistically significant difference in DNA quantity, quality, or STR success between petrous bones and metacarpals III [136]. Sampling strategy should depend on the condition and context of the remains. Metacarpals, being small, are often lost in commingled remains and are more susceptible to degradation with longer PMI [44]. For archaeological samples, petrous bones are recommended, while metacarpals III are preferable in forensic cases with well-preserved remains due to easier handling and lower contamination risk.

Despite the clear advantages of the petrous bone in yielding high amounts of endogenous DNA, its sampling remains highly invasive and technically demanding. The petrous bones are embedded deep within the cranial base and cannot be accessed through the foramen magnum without significant disruption. As there are only two petrous bones per individual, removal of even one can result in major skull damage or fragmentation, particularly in fragile or poorly preserved remains. Accessing the petrous bone in an intact cranium typically requires disarticulation of the temporal bone via cutting through the cranial vault or base, thereby compromising craniometric landmarks essential for anthropological analyses—especially in the study of rare ancient populations [137]. This destructive approach also poses ethical concerns in forensic contexts, where the preservation of remains and respect for the deceased is vital.

Two primary technical approaches have been developed to mitigate these challenges. Pilli et al. [129] utilized a vertical cutting technique with a plunge-cut saw to extract the petrous bone through the foramen magnum. While effective, this method is still relatively invasive and requires substantial equipment and expertise. In contrast, Gonzalez et al. [54] proposed a minimally destructive method involving mechanical surface abrasion and targeted drilling to collect bone powder from the cochlea. This technique allows for near-complete preservation and repositioning of the petrous bone post-sampling.

An alternative method—the Cranial Base Drilling Method (CBDM), introduced by Sirak et al. [138]—accesses the osseous labyrinth through the cranial base without removing the temporal bone. This preserves critical morphological structures and significantly reduces overall damage. Although DNA yields obtained using CBDM are reported to be 0.04 to 2.1 times lower than those from traditional petrous bone sampling, they remain superior to yields from non-petrous postcranial bones.

Recent findings highlight the utility of tooth cementum as a reliable and minimally invasive source of aDNA, offering performance comparable to, and in some cases exceeding, that of petrous bones. While the petrous portion of the temporal bone is widely regarded as the gold standard due to its dense structure and high DNA yield, its destructive sampling poses ethical and curatorial challenges, particularly when dealing with valuable or irreplaceable historical specimens. In contrast, tooth cementum allows for nondestructive DNA extraction, preserving the physical integrity of the specimen for future morphological or isotopic analyses. Studies have shown that when DNA is preserved, tooth cementum achieves STR typing success rates equivalent to those of petrous bones, likely due to reduced DNA degradation during extraction and lower susceptibility to contamination. This makes it particularly advantageous in forensic contexts, where physical preservation of remains is ethically important and contamination must be minimized. Moreover, in well-preserved teeth, cementum has proven to be as effective as petrous bone in recovering genetic profiles even from ancient samples, such as those from the Bronze Age and Viking period. Consequently, tooth cementum represents a practical and ethically favorable alternative for genetic analysis, especially in cases where preservation, contamination risk, or sample destructiveness are key considerations [139,140].

## 7. Discussion

Significant advancements have been made since bones were first recognized as a viable source of DNA for postmortem identification. From the pioneering genotyping of archaeological samples such as the quagga—extinct for over 140 years—to modern breakthroughs in extracting DNA from highly mineralized bone tissues, the field of forensic genetics has evolved substantially. Sampling strategies have become more refined, with growing evidence supporting the use of small skeletal elements rich in trabecular bone due to their higher DNA yield and more efficient STR amplification. These approaches enable faster and more cost-effective identification of human remains.

Recent studies have highlighted significant interskeletal and intraskeletal variability in DNA preservation across different anatomical regions [75,78,89,130,136]. Dense bones, such as the petrous part of the temporal bone, have consistently demonstrated superior DNA preservation in long PMI scenarios [81], while smaller trabecular-rich bones, such as phalanges and metacarpals, are frequently missing in cases of shallow burials or extensive decomposition [141].

However, if environmental conditions allow for the preservation of trabecular bones, they can represent an optimal choice for DNA sampling. This was exemplified by the Konfin II Second World War mass grave site (PMI ~80 years), where both metacarpals and petrous bones yielded comparable DNA quantity and quality, contrary to earlier findings that emphasized the superiority of petrous bones [78,81].

Preservation trends observed in long PMI samples are mirrored in more recent remains. Studies involving fresh bones with PMIs ranging from 1 month to 21 years have consistently shown that trabecular bones yield higher amounts of DNA than cortical bones, irrespective of PMI length [77,79].

## 8. Conclusions

Taken together, these findings suggest that PMI alone does not critically determine DNA yield in forensic samples. Instead, bone microstructure appears to be the primary factor, with trabecular bones generally providing higher DNA quantities than cortical bones, even in longer PMI, if preservation conditions are favorable.

It is essential to note that the PMI plays a key role in determining which bones remain available for sampling. In archaeological contexts, where PMI is very long, trabecular bones typically degrade and are absent, necessitating reliance on dense cortical bones for analysis. However, when environmental conditions favor preservation, even fragile skeletal elements can be viable sources of DNA.

Accordingly, we recommend that forensic sampling strategies incorporate small skeletal elements such as hand and foot bones alongside already validated elements like the petrous bone. Including a broader range of bone types may enhance identification efficiency in DVI and missing persons cases. Moreover, it minimizes the need for repeated re-sampling and re-labeling, aligning with the third ISFG recommendation [44]. In situations where small skeletal elements are absent—particularly in cases of shallow burial or exposure—dense bones such as the petrous part of the temporal bone remain the most reliable alternative for successful DNA recovery.

## Figures and Tables

**Table 1:** Comparative Overview of Studies Supporting vs. Challenging Existing Bone Sampling Guidelines for DNA Analysis.

	Studies Evaluating Skeletal Sample Selection	
	Studies Supporting Current Guidelines	Recent Studies Challenging Current Guidelines
Number of Anatomically Different Bones	15–20	20–55
Number of Samples	1021–24,656	144–3868
PMI	4–59 years	Months to 75 years
Top-Ranked Skeletal Elements	Rib, Femur, Tibiae, Teeth	Phalanges, Carpals, Metacarpals, Metatarsals, Petrous bone, Teeth
Authors	Edson et al., 2004; [49] Leney, 2006; [76] Miloš et al., 2007 [50]	Mundorff et al., 2009; [51] Mundorff and Davoren, 2014; [73] Emmons et al., 2020; [74] Zupanc et al., 2021; [75] Otagiri et al., 2024; [77] Inkret et al., 2025; [78]
